# CD19 Cell Count at Baseline Predicts B Cell Repopulation at 6 and 12 Months in Multiple Sclerosis Patients Treated with Ocrelizumab

**DOI:** 10.3390/ijerph18158163

**Published:** 2021-08-02

**Authors:** Gianmarco Abbadessa, Giuseppina Miele, Paola Cavalla, Paola Valentino, Girolama Alessandra Marfia, Elisabetta Signoriello, Doriana Landi, Chiara Bosa, Marco Vercellino, Antonio De Martino, Rosanna Missione, Maddalena Sparaco, Luigi Lavorgna, Giacomo Lus, Simona Bonavita

**Affiliations:** 1 Department of Advanced Medical and Surgical Sciences, University of Campania Luigi Vanvitelli, 80131 Naples, Italy; gianmarco.abbadessa@unicampania.it (G.A.); giuseppina.miele@unicampania.it (G.M.); elisabetta.signoriello@unicampania.it (E.S.); rosanna.missione@unicampania.it (R.M.); maddalena.sparaco@unicampania.it (M.S.); luigi.lavorgna@policliniconapoli.it (L.L.); giacomo.lus@unicampania.it (G.L.); 2 Multiple Sclerosis Center, AOU Città della Salute e della Scienza di Torino, 10147 Turin, Italy; paola.cavalla@unito.it (P.C.); chiara.bosa@unito.it (C.B.); marco.vercellino@unito.it (M.V.); 3 Institute of Neurology, University “Magna Graecia”, 88100 Catanzaro, Italy; p.vale@unicz.it (P.V.); antonio.demartino001@studenti.unicz.it (A.D.M.); 4 Multiple Sclerosis Clinical and Research Unit, Department of Systems Medicine, Tor Vergata University, 00133 Rome, Italy; marfia@med.uniroma2.it (G.A.M.); doriana.landi@unicamillus.org (D.L.)

**Keywords:** multiple sclerosis, ocrelizumab, schedule, B cell, CD20, CD19, kinetics

## Abstract

Background: The kinetics of B cell repopulation in MS patients treated with Ocrelizumab is highly variable, suggesting that a fixed dosage and time scheduling might be not optimal. We aimed to investigate whether B cell repopulation kinetics influences clinical and radiological outcomes and whether circulating immune asset at baseline affects B cell repopulation kinetics. Methods: 218 MS patients treated with Ocrelizumab were included. Every six months we collected data on clinical and magnetic resonance imaging (MRI) activity and lymphocyte subsets at baseline. According to B cell counts at six and twelve months, we identified two groups of patients, those with fast repopulation rate (FR) and those with slow repopulation rate (SR). Results: A significant reduction in clinical and radiological activity was found. One hundred fifty-five patients had complete data and received at least three treatment cycles (twelve-month follow-up). After six months, the FR patients were 41/155 (26.45%) and 10/41 (29.27%) remained non-depleted after twelve months. FR patients showed a significantly higher percentage of active MRI scan at twelve months (17.39% vs. 2.53%; *p* = 0,008). Furthermore, FR patients had a higher baseline B cell count compared to patients with an SR (*p* = 0.02 and *p* = 0.002, at the six- and twelve-month follow-ups, respectively). Conclusion: A considerable proportion of MS patients did not achieve a complete CD19 cell depletion and these patients had a higher baseline CD19 cell count. These findings, together with the higher MRI activity found in FR patients, suggest that the Ocrelizumab dosage could be tailored depending on CD19 cell counts at baseline in order to achieve complete disease control in all patients.

## 1. Introduction

Ocrelizumab (Ocrevus^®^) was approved for the treatment of adults with relapsing (RMS) [1] and primary progressive multiple sclerosis (PPMS) [2]. It is a humanized anti-CD20 cell-depleting antibody. CD20 is a cell surface antigen expressed on the surface of pre-B, mature and also memory B lymphocytes [3].

As per other anti-CD20 drugs, the effector mechanisms of Ocrelizumab are complement-dependent cytotoxicity, antibody-dependent cellular cytotoxicity and antibody-dependent cellular phagocytosis [4]. 

The therapeutic efficacy of anti-CD20 cell-depletive drugs is related to the reduction in the B cell antigen-presenting activity and antigen nonspecific immune functions [4,5]. 

Ocrelizumab is administered on a 6-month fixed interval schedule, for both relapsing and progressive MS. However, B cell kinetics in MS patients treated with Ocrelizumab is highly variable, suggesting that a fixed time scheduling might not be optimal [6]. Further studies have revealed the presence of clinical and biological markers able to predict B cell kinetics in patients treated with anti-CD20 drugs [7].

This body of evidence raised the following question: why does the drug need to be administered at fixed intervals and fixed dosage but not tailored on measurable predicting markers of B cell repopulation?

Given the extreme inter-individual variability in the disease course and the individual factors influencing B-cell repopulation, Ocrelizumab therapy with fixed dosage and schedule may actually increase the risk of insufficiently effective treatment or overtreatment. Accordingly, reliable biomarkers need to be identified for a better stratification of patients undergoing Ocrelizumab treatment to tailor therapy based on clinical and laboratory parameters. 

Bearing in mind the aforegoing, we explored the effectiveness of Ocrelizumab in a cohort of MS patients and investigated whether B cell repopulation kinetics influences clinical and radiological outcomes. Furthermore, we investigated whether circulating immune asset at baseline affects B cell repopulation kinetics.

## 2. Materials and Methods

We designed a multicenter, retrospective study based on prospectively collected data involving 5 Italian MS centers, namely, MS Center, Tor Vergata University, Rome, MS Center, University “Magna Graecia”, Catanzaro, MS Center, AOU Città della salute e della Scienza di Torino, Turin, and MS Center, University of Campania “Luigi Vanvitelli”, Naples. 

We included MS patients who started Ocrelizumab treatment according to clinical practice between January 2018 and October 2020 and completed at least a six-month follow up. We excluded patients who had been previously treated with other anti-CD20 drugs (i.e., Rituximab) to avoid the cumulative effects of anti-CD20 drug administration on study outcomes. MS course was defined as RMS, Secondary Progressive MS (SPMS) (with or without disease activity) or PPMS (with or without disease activity) [8]. Demographic data (age and sex) and MS history (MS onset date, age at onset, disease duration, MS phenotype, previous disease modifying therapy (DMT), number of relapses twelve months and two years before Ocrelizumab treatment to determine the annualized relapse rate (ARR)) were collected.

Clinical, radiological and biological outcomes during Ocrelizumab treatment were assessed at the time of therapy initiation and every six months thereafter. Clinical evaluation was performed by assessing EDSS and the number of relapses to determine the ARR. Furthermore, we collected radiological data, such as new T2 lesions and T1 gadolinium enhancing (gd+) lesions.

We collected data to assess circulating immune asset before Ocrelizumab administration and every six months (lymphocyte subpopulations by flow cytometry as CD19+, CD4+, CD8+ and CD16+CD56+). As in previous studies, CD19 cell count was used as a surrogate of CD20 cell count.

Ocrelizumab was administered according to FDA and EMA schedule approval for MS: two 300 mg IV infusions two weeks apart, followed by 600 mg every six months or every 24 weeks. Before each infusion, clinical and instrumental evaluations were performed along with blood samples.

NEDA-3 (no evidence of disease activity) status was calculated at the twelve-month follow-up. NEDA-3 was defined as absence of relapses, 12 weeks confirmed disability worsening (CDP) and magnetic resonance (MRI) activity. The CDP was defined as (i) ≥1.5-point increase, if EDSS = 0 at baseline, (ii) ≥1.0-point increase, if EDSS = 0.5–5 at baseline, or (iii) ≥0.5-point increase, if EDSS > 5.0 at baseline [9]. Relapse was defined as new or recurrent neurological symptoms not associated with fever or infection lasting for ≥24 h and accompanied by new neurological signs [10]. Radiological activity was defined as the appearance of T1 gadolinium, or new or enlarging T2-hyperintense lesions, compared with the previous scan [11].

According to B cell count, patients were divided into two groups, fast (FR) and slow repopulation rate (SR), each based respectively on whether there was a reappearance or otherwise of CD19 at the six- and twelve-month follow-ups. In line with previous studies, B cell reappearance was defined when CD19 cells reached 1% of lymphocyte count [7,12,13].

### Statistical Analysis

The characteristics of the study population were presented using descriptive statistics. Mean and standard deviation (SD) values were calculated for continuous variables, while frequencies were reported for categorical variables.

All continuous data presented in Table 1 were tested for normality using the Shapiro–Wilk test. Differences in demographic and clinical features at baseline between disease phenotype subgroups were calculated using analysis of variance, Fisher exact test and U Mann–Whitney non-parametric test, when appropriate.

In the whole population, in the disease phenotype subgroups and in FR and SR patients, the effectiveness of treatment was calculated using the Wilcoxon test for paired samples, analyzing the ARR, EDSS and active lesions on MRI at baseline and after six- and twelve-month periods of treatment.

The differences between total lymphocytes, CD3, CD19, CD4, CD8 and CD16CD56 cells at baseline and after both six- and twelve-month periods of treatment were evaluated only in patients with complete data using the analysis of variance for repeated measures.

Differences in treatment effectiveness between FR and SR were analyzed comparing NEDA-3 and radiological activity (as defined in the methods section) at twelve months by means of the Chi square test. 

The U Mann–Whitney non-parametric test for independent sample was used to compare CD19+, CD4+, CD8+ and CD16+CD56+ cell counts and percentages at baseline in the two populations (FR and SR). A receiver operating curve (ROC) analysis was performed to determine a cut-off of the CD19 cell level at baseline identifying patients with fast or slow CD19 repopulation. Statistical analyses were performed using Stata (StataCorp. 2019.Stata Statistical Software: Release 16. StataCorp LLC, College Station, TX, USA). Statistical significance was accepted when *p* value < 0.05.

## 3. Results

Two-hundred eighteen MS patients were enrolled; clinical and demographic features at treatment initiation for the whole population, RMS (*n* = 127), SPMS (*n* = 43) and PPMS (*n* = 48) are summarized in Table 1.

**Figure 1:**
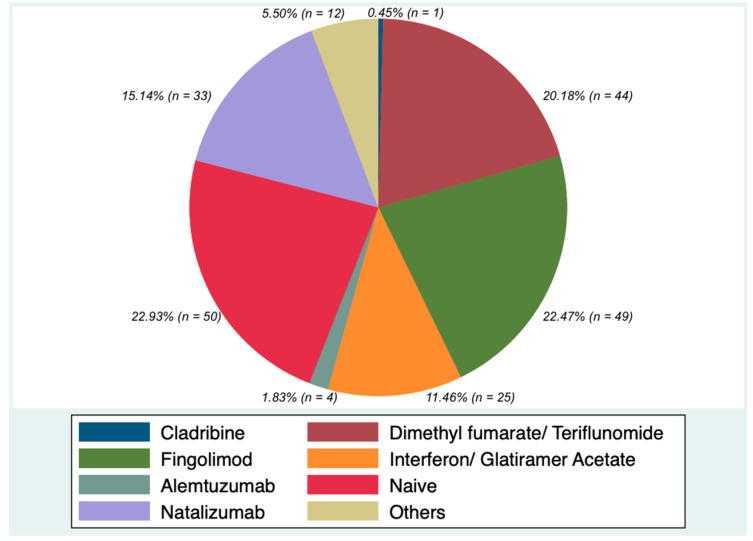
Frequency of DMTs before Ocrelizumab in the whole sample.

A total of 51 out of 218 (23.04%) patients was treatment naive, while 167/218 (76,95%) patients switched from other treatments (Figure 1 shows the frequency of previously used DMTs in all patients).

Adverse events and reasons for switch to Ocrelizumab are reported in Table S1–S3, in Supplementary Material.

At enrollment, 218 patients had received at least 2 treatment cycles (six-month follow-up) and 155 patients had received at least 3 treatment cycles (twelve-month follow-up).

Therefore, when we compared clinical and radiological data at baseline with those at 6 months, we were considering 218 patients. Conversely, when we compared clinical and radiological data at baseline with those at twelve months, we were taking into consideration 155 patients who had the twelve-month follow-up.

MRI scans at the six- and twelve-month follow-ups were available, respectively, for 187 and 113 patients.

### 3.1. Ocrelizumab Effectiveness in the Whole Sample, Secondary Progressive, Primary Progressive and Relapsing Remitting Patients

We firstly analyzed the effectiveness of treatment in the whole cohort. We found a significant reduction in the ARR (*p* < 0.001) and in active lesions at the six- and twelve-month follow-ups (*p* < 0.001) and EDSS stabilization.

Then, Ocrelizumab effectiveness was evaluated in RMS, PPMS and SPMS patients.

RMS patients showed a significant reduction in the ARR (*p* < 0.001) and of active lesions at the six- and twelve-month follow-ups (*p* < 0.001) and EDSS reduction at the twelve-month follow-up, but not statistically significant. NEDA-3 was achieved in 69.49% of RMS patients by the twelve-month follow-up. 

SPMS showed a significant reduction in active lesions at six months (*p* = 0.0253); a reduction in the ARR and active lesions at twelve months was found, but not statistically significant.

In PPMS, a significant reduction in the ARR (*p* = 0.008), a reduction in active lesions at the six- (*p* = 0.0028) and twelve-month follow-ups (*p* = 0.0458) and a positive trend for reduction in EDSS was found. However, for the reduction in active lesions at twelve months, the statistical significance is borderline. Table 2 summarizes the effectiveness of Ocrelizumab in the overall population and in the different disease courses.

### 3.2. Ocrelizumab Effectiveness in Patients with Fast and Slow Repopulation Rate

**Figure 2:**
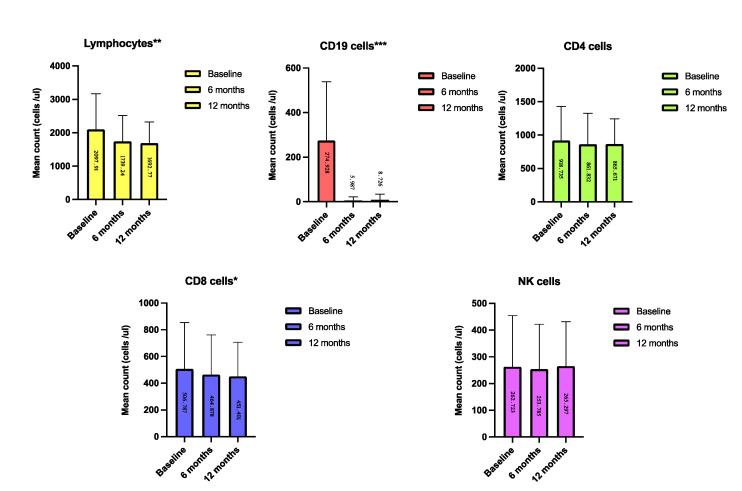
Absolute lymphocyte and lymphocyte subset counts at baseline, after six and twelve months of Ocrelizumab treatment (mean counts). For all the immune cells, 155 patients had a twelve-month follow-up and were included in the analysis of variance for multiple comparison; a significant reduction in mean absolute number of lymphocytes, CD8 T cells and CD19 cells was revealed. * *p* < 0.05; ** *p* < 0.01; *** *p* < 0.001.

Over the twelve-month treatment period, in patients with a complete follow up and complete data (*n* = 155), a significant reduction in CD19 cells was revealed (12.94% at baseline vs. 0.45% after twelve months (*p* = 0.001)); we also found a significant decrease in total lymphocytes (*p* = 0.0012) and CD8 cell count (*p* = 0.01), but not CD4 cell count (details in Figure 2).

After six months, the FR patients were 41/155 (26.45%) and, among them, 10/41 (29.27%) were not depleted after twelve months. In the SR group at six months, only 12.28% was not depleted after twelve months (*p* = 0.013). 

Table 3 shows treatment effectiveness in FR and SR at the six- and twelve-month follow-ups; SR patients showed a significant reduction in the ARR (*p* < 0.001) and a reduction in active lesions at six- (*p* < 0.001) and twelve-months of treatment (*p* < 0.001). FR patients showed a significant reduction in the ARR (*p* = 0.0012) and active lesions at six and twelve months (*p* = 0.0325 and *p* = 0.0143, respectively). A trend for stabilization of EDDS was found in both groups. 

Comparing FR and SR patients for Ocrelizumab effectiveness, FR patients at six months showed a significantly higher percentage of active MRI scan than at twelve months (17.39% vs. 2.53%; *p* = 0.008). No difference was found in NEDA-3 status between the two subgroups.

### 3.3. Predictors of B Cell Repopulation

Comparing baseline CD19 cell counts in FR and SR at six months, FR had a significantly higher CD19 cell count (15.76 ± 9.23% and 12.45 ± 7.29%, respectively; *p* = 0.02). The ROC curve analysis determined 12% of CD19 cells at baseline as the cut-off point, generating the best combination of sensitivity and specificity, for B cells reappearance at six months (AUC, 0.62 (0.52–0.73)). 

FR at twelve months had a significantly higher CD19 cell count at baseline, compared to SR at twelve months (18.51 ± 9.68% and 12.2 ± 6.35%, respectively; *p* = 0.002). The ROC curve analysis determined 14% of CD19 cells at baseline as cut-off point, generating the best combination of sensitivity and specificity, for B cell reappearance at twelve months (AUC, 0.71 (0.58–0.83)). 

Therefore, in patients with higher CD19 cell count at baseline, CD19 were not completely depleted after six and twelve months of treatment.

No difference was found in CD4+, CD8+ and CD16+CD56+ cell counts at baseline in the two populations. Comparison of total lymphocytes and lymphocyte subset count at baseline between patients with fast and slow repopulation rate at six and twelve months is displayed in Table 4 and Table 5, respectively.

## 4. Discussion

This is the largest retrospective observational study investigating not only clinical and radiological outcomes but also laboratory ones of MS patients treated with Ocrelizumab. We further explored the possible influence of circulating immune asset on these outcomes. 

In phase III trials (OPERA I and OPERA II) [1], a total of 1656 RMS patients were included. In the Ocrelizumab group, the ARR reduction was 46% and 47%, in OPERA I and OPERA II, respectively.

Our study confirms the real-life effectiveness of Ocrelizumab in RMS; we observed a significant reduction in the ARR (82.15%; 0.56 vs. 0.1) and radiological activity at six and twelve months (85.8% and 96.8%, respectively) in RMS. As generally found in observational studies [14], our sample also showed that the ARR reduction was higher than the one observed in clinical trials; in actual fact, the pre-treatment ARR of our patients was 0.56 (±0.78 SD) vs. 1.31 (±0.65 SD) in the clinical trial population. Furthermore, another possible explanation for this discrepancy could depend on the lower relapse frequency assessment in the observational studies [14]. The proportion of RMS patients that reached NEDA-3 in our cohort (69.5%) was slightly higher than in the phase III trial (47.9%); however, the shorter-term follow-up should be considered [15].

As for progressive phenotypes, in line with clinical trial results, our study revealed good effectiveness of Ocrelizumab in disease activity (reduction in the ARR and active lesions in MRI).

Regarding the effect of Ocrelizumab on circulating immune asset at the six- and twelve-month follow-ups, a significant reduction in mean lymphocyte cell count was found.

Our findings also revealed a slight but significant reduction in CD8 cells after six- and twelve-month periods of therapy. A small subset of T cells (both CD4 and CD8 cells) also expresses CD20 [16] and recent studies have revealed that Ocrelizumab efficiently depletes this subset of cells [16]. These findings could explain the reduction in CD8 cells observed in our study. However, the effect of CD8+CD20+ cell depletion on Ocrelizumab effectiveness is still unexplored.

Overall, beside the B cells, there were only minor changes in CD8 T cells and no effect on any other cell types. However, the patients showed improvement in disease activity. Although apparently conflicting, we should consider that among CD8 lymphocytes there are different cell subtypes and only some of them are involved in the immune-mediate inflammation. B cell depletion could be associated with a change in CD8 cell profile [17]. Our analysis did not make any distinction between CD8 naïve cells, CD8 pro-inflammatory effector cells and CD8 regulatory cells. Therefore, we cannot exclude that, despite the slight reduction in CD8 lymphocytes, B cell depletion greatly affected CD8 cell profiles, dampening their pro-inflammatory role in MS.

With regard to B cell kinetics, our results show the depletion of B cells after six- and twelve-months in most of the patients. However, 26% of them had an incomplete depletion at six months that was confirmed in 1/3 of them at twelve months. 

CD20-depleting drugs induce CD20 suppression in peripheral blood serum [4,5]. 

The therapeutic efficacy of anti-CD20 cell-depletive drugs is related to the reduction in the B cell antigen-presenting activity and antigen nonspecific immune functions [4,5]. 

Further, Kletzl and colleagues showed that higher Ocrelizumab exposure leads to greater B-cell depletion and guarantees a greater risk reduction in CDP [18].

These data raise concern that incomplete B cell depletion could negatively affect clinical outcomes of MS patients treated with anti-CD20 drugs. 

Although several studies reported that B cell repopulation is associated with an increase in disease activity in neuromyelitis optica spectrum disorder [19], in a recent observational study in MS patients treated with Ocrelizumab, no differences were reported between FR and SR, in terms of clinical outcomes [7]. However, in the study, the authors did not consider radiological outcomes in their analysis and, beside which, their sample size was smaller than ours. 

Our results show a significant higher proportion of active MRI scans at twelve months in FR patients, compared to SR patients, suggesting that Ocrelizumab needs to be administered based on peripheral B cell count monitoring.

Indeed, since there may be high inter-individual variance in repopulation rate, personalized dose regimens could be necessary to optimize treatment response. Therefore, in order to identify measurable predictors of early B cell reappearance, we analyzed circulating immune biomarkers associated with the FR condition. We found that B cell count at baseline could be a predictor of early reappearance of B cells at six and twelve months. In actual fact, FR patients (both at six and twelve months) had higher B cell counts at baseline, compared to SR patients. In this perspective, we identified the cut-off of CD19 cells at baseline that could identify patients with incomplete depletion, therefore needing Ocrelizumab posology adaptation.

Our findings show a cut-off of 12% of CD19 cells at baseline for the occurrence of B cell repopulation at six months. Conversely, a cut-off of 14% of CD19 cells at baseline was revealed for the occurrence of B cell repopulation at twelve months. 

Therefore, we could hypothesize that patients with a B cell proportion at baseline between 12% and 14% could receive only a higher starting dose. Conversely, those patients with a B cell proportion higher than 14% could receive a higher dosage at the first two cycles. However, a further possible approach could be to shorten the interval between the administrations. Therefore, patients with a B cell proportion at baseline between 12% and 14% could shorten the interval between the first two cycles and those patients with a B cell proportion at baseline higher than 14% could shorten the interval between the first two cycles and between the second and the third ones.

A higher B cell number at baseline could reflect a trend towards a faster B cell reappearance with faster differentiation of B cells into inflammatory subsets, therefore a higher disease activity. Aligned, our data revealed that the baseline B cell number could predict B cell reappearance that, in turn, is associated with a higher disease activity. Therefore, Ocrelizumab dosage could be a critical factor for complete depletion and could be tailored based on the B cell number at baseline to obtain disease control in all patients. However, considering that our findings are limited to the first twelve-month follow-up, this hypothesis is not generalizable to the dosage for cycles following the second one.

In our population, early B cell reappearance was associated with higher disease activity. Despite the fact that a recent study demonstrated that the delay in dosing of Ocrelizumab due to COVID-19 pandemic was an independent predictor of B cell repopulation, the study findings do not show clinical consequences in delaying Ocrelizumab [20]. Nonetheless, the small number of patients enrolled could have greatly influenced this result.

Based on our results, we suggest that, at initiation of Ocrelizumab, the dosage could be adapted based on B cell count at baseline to achieve a complete B cell depletion.

Lastly, we would also highlight the detrimental effect of anti-drug antibodies (ADA) that could greatly reduce the effect and efficacy of biopharmaceuticals. The introduction of biopharmaceuticals in clinical practice has improved treatment strategy and clinical care of patients with MS [20]. The immunogenic properties of these agents can lead to immunogenic reaction and the production of neutralizing ADAs [21]. Neurologists managing MS patients are used to test ADA against Interferon and Natalizumab as an integrated part of clinical practice. For patients treated with Natalizumab, ADAs testing is usually performed for adverse events to the infusion or for ineffectiveness [21]. However, in our study, patients treated with Natalizumab before Ocrelizumab initiation did not perform ADAs testing, as they had switched mainly for safety reasons. ADA testing for Interferon is no longer performed as, currently, there are several therapeutic options and clinical ineffectiveness is quite enough to drive drug withdrawal. Concerning anti-CD20 agents, a previous study reported the presence of ADAs against Rituximab in 37% of RMS and 26% of progressive MS patients [22]. There was a significant association between both presence and titers of ADA and partial B-cell depletion, but not with adverse events or efficacy. Conversely to Rituximab, Ocrelizumab is a humanized monoclonal antibody [1]. Humanization might reduce the immunogenic properties, therefore ADA production [23]. Indeed, a reduced risk of ADAs formation with humanized antibodies, such as ocrelizumab, compared with chimeric antibodies, is expected. Recent findings from phase III Interferon-β-1a-controlled trials of Ocrelizumab revealed that the incidence of treatment-emergent ADA post-Ocrelizumab start was 0.2–0.5 per cent [23]. No patient performed ADA testing in our population, as it is not a common clinical practice; therefore, we cannot conclude whether and how the emergence of ADA in our population might have influenced clinical and biological findings. Adding ADA testing for SR patients in clinical practice might be helpful for treatment optimization.

This study has several limitations due to the retrospective and observational design; the higher proportion of male patients in the SP group could be due to an enrollment bias. However, although this was a retrospective study, the MS centers involved are used to collecting data on Ocrelizumab treated patients prospectively and with fixed intervals. Furthermore, despite the absence of standardized MRI protocols could be a bias for radiological outcomes, non-standardized MRI protocols tend to underestimate disease activity rather than overestimate it [24].

## 5. Conclusions

In conclusion, we found that CD19 cell count at baseline could influence B cell kinetics; indeed, our findings revealed that a considerable proportion of MS patients did not achieve a complete CD19 cell depletion and these patients had a higher baseline CD19 cell count. These findings, together with the higher MRI activity found in FR patients, suggest that Ocrelizumab dosage could be tailored based on CD19 cell count at baseline to achieve a complete disease control in all patients.

These results need corroboration in larger studies and could be useful in clinical practice to tailor treatment dosage and schedules.

## Figures and Tables

**Table 1:** Whole population’s demographic and clinical characteristics. Data are reported using standard descriptive statistics, mean ± standard deviation (sd), in the case of numerical variables, and absolute frequencies and percentages for categorical factors. All continuous data (mean ± standard deviation) are not normally distributed.

Demographic and Clinical Characteristics	Descriptive Statistics	Overall (218)	SP (43)	PP (48)	RR (127)	*p* Value
Age (y)	Mean (SD)	42.01 (13.45)	50.18 (8.62)	48.68 (8.59)	40.87 (12.43)	<0.001
Sex (female)	Number (%)	124 (56.88)	46 (37.09)	67 (54.03)	79 (64.70)	0.0042
Age at disease onset (y)	Mean (SD)	31.39 (12)	35.89 (13.65)	32.12 (13.36)	36.92 (19.15)	0.2074
Disease duration (y)	Mean (SD)	18.75 (27.19)	25.33 (33.48)	21.10 (25.79)	25.81 (39.11)	0.6969
ARR previous year	Mean (SD)	0.56 (0.78)	0.13 (0.34)	0.19 (0.39)	0.82 (0.87)	<0.001
Baseline EDSS	Mean (SD)	4.59 (2.05)	5.58 (1.47)	5.83 (1.44)	3.41 (1.62)	<0.001
Wash out (months)	Mean (SD)	10.06 (21.31)	4.77 (7.59)	10.41 (16.72)	8.13 (15.05)	0.312
Treatment duration (y)	Mean (SD)	1.30 (0.87)	1.93 (0.88)	1.98 (0.80)	1.81 (0.80)	0.351
Age at Ocrelizumab start (y)	Mean (SD)	40.70 (13.64)	48.25 (8.89)	46.70 (8.72)	39.14 (12.49)	<0.001

Note: SP, secondary progressive; PP, primary progressive; RR, relapsing remitting; ARR, annualized relapse rate; EDSS, expanded disability status scale.

**Table 2:** Ocrelizumab effectiveness in overall patients, secondary progressive, primary progressive and relapsing remitting patients. Data are reported using mean ± standard deviation (sd).

Clinical and Radiological Features	Overall Population	*p* Value	SP	*p* Value	PP	*p* Value	RR	*p* Value
ARR pre therapy	0.56 (0.78)	<0.001	0.13 (0.34)	0.414	0.19 (0.39)	0.008	0.82 (0.87)	<0.001
ARR 12 months post therapy	0.10 (0.32)		0.07 (0.27)		0.02 (0.14)		0.14 (0.37)	
EDSS baseline	4.37 (1.92)	0.736	5.58 (1.47)	0.176	5.83 (1.44)	0.490	3.41 (1.62)	0.177
EDSS six months	4.33 (1.97)		5.7 (1.54		5.81 (1.47)		3.42 (1.68)	
EDSS baseline	4.37 (1.92)	0,929	5.58 (1.47)	0.113	5.83 (1.44)	0.919	3.41 (1.62)	0.340
EDSS twelve months	4.16 (1.99)		5.6 (1.67)		5.40 (1.79)		3.47 (1.65)	
Active lesions baseline	0.28 (0.49)	<0.001	0.16 (0.37)	0.0253	0.35 (0.59)	0.0028	0.29 (0.47)	<0.001
Active lesions 6 months	0.04 (0.26)		0 (0)		0.03 (0.17)		0.06 (0.30)	
Active lesions baseline	0.28 (0.49)	<0.001	0.16 (0.37)	0.0833	0.35 (0.59)	0.0458	0.29 (0.47)	<0.001
Active lesions 12 months	0.009 (0.09)		0 (0)		0 (0)		0.01 (0.11)	
NEDA-3							69.49%	

*Note:* SP, secondary progressive; PP, primary progressive; RR, relapsing remitting; ARR, annualized relapse rate; EDSS, expanded disability status scale; NEDA-3 (no evidence of disease activity).

**Table 3:** Ocrelizumab effectiveness in patients with fast and slow repopulation rate at six months. Data are reported using mean ± standard deviation (sd).

Clinical And Radiological Features	FR (*n* = 41)	*p* Value	SR (*n* = 114)	*p* Value
ARR pre therapy	0.40 (0.72)	0.0012	0.61 (0.80)	<0.001
ARR 12 months post therapy	0.09 (0.35)		0.12 (0.33)	
EDSS baseline	4.59 (1.74)	0.705	4.23 (1.95)	0.990
EDSS 6 months	4.51 (1.74)		4.23(2.00)	
EDSS baseline	4.59 (1.74)	0.9127	4.23 (1.95)	0.6454
EDSS 12 months	4.21 (1.80)		4.18 (2.03)	
Active lesions baseline	0.31 (0.54)	0.0195	0.25 (0.45)	<0.001
Active lesions 6 months	0.06 (0.33)		0.04 (0.23)	
Active lesions baseline	0.31 (0.54)	0.0143	0.25 (0.45)	<0.001
Active lesions 12 months	0.04 (0.20)		0 (0)	

Note: FR, fast repopulator; SR, slow repopulator; ARR, annualized relapse rate; EDSS, expanded disability status scale.

**Table 4:** Comparison of total lymphocytes and lymphocyte subsets at baseline between patients with fast and slow repopulation rate at six months. Data are reported using mean ± standard deviation (sd).

Immunological Asset	FR (*n* = 41)	SR (*n* = 114)	*p* Value
Lymphocytes (mean, sd)	2027.2 (1066.63)	2104.441 (1032.16)	0.60
CD4 cells (mean, sd)	937.02 (617.97)	918.23 (498.34)	0.61
%CD4 cells (mean, sd)	42.32 (10.66)	43.56 (10.82)	
CD8 cells (mean, sd)	468.18 (286.19)	522.40 (356.25)	0.92
%CD8 cells (mean, sd)	24.44 (8.35)	24.49 (7.39)	
CD19 cells (mean, sd)	349.73 (373.37)	256.42 (225.27)	0.02
%CD19 cells (mean, sd)	15.76 (9.23)	12.45 (7.29)	
CD16CD56 cells (mean, sd)	241.33 (117.76)	274.70 (216.06)	0.61
%CD16CD56 cells (mean, sd)	16.26 (15.52)	16.82 (18.22)	

Note: FR, fast repopulator; SR, slow repopulator.

**Table 5:** Comparison of total lymphocytes and lymphocyte subsets at baseline between patients with fast and slow repopulation rate at twelve months. Data are reported using mean ± standard deviation (sd).

Immunological Asset	FR (*n* = 10)	SR (*n* = 145)	*p* Value
Lymphocytes (mean, sd)	2344.28 (1274.75)	2092.61 (1112.69)	0.38
CD4 cells (mean, sd)	1130.57 (630.84)	897.44 (537.58)	0.07
%CD4 cells (mean, sd)	44.58 (8.81)	42.01(10.84)	
CD8 cells (mean, sd)	517.89 (329.51)	517.59 (383.66)	0.75
%CD8 cells (mean, sd)	20.81(8.56)	24.70 (7.62)	
CD19 cells (mean, sd)	510.69 (502.71)	255.72 (230.40)	0.0025
%CD19 cells (mean, sd)	18.51 (9.68)	12.20 (6.35)	
CD16CD56 cells (mean, sd)	236 (149.26)	284.80 (219.60)	0.29
%CD16CD56 cells (mean, sd)	16.06 (20.70)	15.89 (10.13)	

Note: FR, fast repopulator; SR, slow repopulator.

## Data Availability

The data that support the findings of this study are available on request from the corresponding author. The data are not publicly available due to their containing information that could compromise the privacy of the patients.

## Supplementary Materials

The following are available online at https://www.mdpi.com/article/10.3390/ijerph18158163/s1, Table S1: Demographic and clinical data in patients switching from different DMTs, Table S2: Reasons for switch to Ocrelizumab, Table S3: Adverse events in the whole population.
