# Supplementary material for: CD19 Cell Count at Baseline Predicts B Cell Repopulation at 6 and 12 Months in Multiple Sclerosis Patients Treated with Ocrelizumab
Source: Int J Environ Res Public Health. 2021 Aug 2;18(15):8163. doi: 10.3390/ijerph18158163 (PMC8346113; doi:10.3390/ijerph18158163)
Supplement: Supplementary file 1 [file ijerph-18-08163-s001.zip › ijerph-1278588-supplementary.pdf]

Side effects and reason for switch to Ocrelizumab

Concerning clinical characteristics of patients switching from different DMTs, those switching from natalizumab and alemtuzumab revealed a lower EDSS at baseline compared to those switching from fingolimod and first line therapy (both injective and oral). Switching from fingolimod patients had the highest EDSS at baseline among the DMTs group. Further, patients switching from first line therapies and fingolimod showed a higher disease activity (ARR and active lesions mean count) before Ocrelizumab start compared to those switching from natalizumab. Patients switching from first line therapies showed the highest disease activity among DMTs groups. These findings are in line with reasons for switch to Ocrelizumab in our population. Indeed, reasons for switching were mostly inadequate efficacy for first line therapies, disease progression and inadequate efficacy for fingolimod, safety concerns (high JCV index) for NTZ and scheduled stop for alemtuzumab. Details in table S1 and S2.

Table S1 – Demographic and clinical data in patients switching from different DMTs.

		**Alemtuzumab (4)**	**Cladribine (1)**	**Dimethyl fumarate/ Teriflunomide (44)**	**Fingolimod (49)**	**Interferon/ Glatimer acetate (25)**	**Natalizumab (33)**
**Disease duration (Y)**	Mean (SD)	34.42 (54.37)	20.96 (-)	21.81 (32.78)	23.83 (31.61)	35.43 (43.48)	14.71 (18.56)
**ARR previous year**	Mean (SD)	0.5 (0.57)	0 (-)	0.62 (0.78)	0.53 (0.53)	0.57 (0.50)	0.45 (0.83)
**Baseline EDSS**	Mean (SD)	2.66 (0.28)	4 (-)	4.42 (1.91)	5.09 (1.59)	4.5 (2.22)	3.58 (1.76)
**Active lesions**	Mean (SD)	0 (-)	0 (-)	0.31 (0.56)	0.25 (0.48)	0.30 (0.47)	0.19 (0.40)

Table S2 – Reasons for switch to Ocrelizumab

		Alemtuzumab (4)	Cladribine (1)	Dimethyl fumarate/ Teriflunomide (44)	Fingolimod (49)	Interferon/ Glatimer acetate (25)	Natalizumab (33)
Inadequate Efficacy	Number (%)	0	0	18 (40,90)	13 (26,53)	13 (52)	12 (36,36)
Disease Progression	Number (%)	1 (25)	0	20 (45,45)	33 (67,34)	5 (20)	6 (18,18)
Adverse event/ side effects/ safety*	Number (%)	0	0	4 (9,09)	2 (4,08)	2 (8)	15 (45,45)
Non adherence/ non compliance	Number (%)	0	0	2 (4,54)	1 (2,04)	5 (20)	0
Scheduled stop	Number (%)	3 (75)	1 (100)	0	0	0	0
Others	Number (%)	0	0	0	0	0	0

*Safety for Natalizumab group is referred to a high qualitative detection of antibodies to the polyomavirus JC virus.

Adverse events

Our findings concerning safety data revealed that only 38 patients experienced an adverse event. The most frequent was urinary infection, followed by respiratory infection and infusion related reaction. Details in Table S3.

Table S3 – Adverse events in the whole population

Adverse events	P	%
N of patients with adverse event	38/218	17,43
Infection	27/38	71,05
Urinary infection	13/38	
Respiratory infection	8/38	
Varicella zoster	2/38	
Warts	1/38	
Herpes simplex	2/38	
Viral pericarditis	1/38	
Universalis alopecia	1/38	2,63
Infusion related reaction	6/38	15,79
Tendon Rupture	1/38	2,63
Glycemic increase	2/38	5,26
Ischemic heart attack	1/38	2,63
